# Vocational Profile and Correlates of Employment in People With Schizophrenia: The Role of Avolition

**DOI:** 10.3389/fpsyt.2020.00856

**Published:** 2020-08-27

**Authors:** Mei San Ang, Gurpreet Rekhi, Jimmy Lee

**Affiliations:** ^1^Research Division, Institute of Mental Health, Singapore, Singapore; ^2^North Region & Department of Psychosis, Institute of Mental Health, Singapore, Singapore; ^3^Lee Kong Chian School of Medicine, Nanyang Technological University, Singapore, Singapore

**Keywords:** employment, vocational rehabilitation, occupation, work outcomes, schizophrenia, negative symptoms, Motivation and Pleasure (MAP), Avolition

## Abstract

**Objective:**

Employment was associated with recovery in individuals with schizophrenia. Our study aimed to delineate the vocational profile and investigate factors associated with likelihood of employment in individuals with schizophrenia.

**Materials and Methods:**

276 community dwelling outpatients with schizophrenia were recruited; 274 completed the Positive and Negative Syndrome Scale (PANSS) and Brief Negative Symptom Scale (BNSS). Information on employment status, work outcomes and demographics were collected. Occupation was coded in accordance with the Singapore standard occupational classification. Either BNSS Motivation and Pleasure (MAP) and Emotional Expressivity (EE) or BNSS five-factor (Anhedonia, Asociality, Avolition, Blunted Affect, Alogia) were examined with PANSS factors and demographics in logistic regression with employment status and working full-time as outcome variables.

**Results:**

One-hundred and twenty-seven (46.01%) participants were employed; 65 (51.18%) worked full-time. In the model with BNSS MAP-EE, MAP (*OR*=0.897, CI=0.854-0.941) and presence of physical comorbidity (*OR*=0.533, CI=0.304-0.937) were associated with reduced likelihood of employment; female sex (*OR*=0.286, CI=0.128 - 0.637) was associated with working part-time. In the model with BNSS five-factor, Avolition (*OR*=0.541, CI=0.440-0.666), and PANSS Positive (*OR*=0.924, CI=0.855-0.997) were associated with reduced likelihood of employment; female sex (*OR*=0.289, CI=0.126 - 0.662) and Avolition (*OR*=0.644, CI=0.475 - 0.872) were associated with working part-time.

**Discussion:**

Our study described the vocational profile and correlates of employment in a developed urban Asian country. Negative symptoms, particularly MAP and Avolition, positive symptoms, and physical comorbidity reduced an individual’s likelihood of employment, while female sex and Avolition were associated with working part-time. Efforts to identify and address these factors are necessary to encourage employment in individuals with schizophrenia.

## Introduction

Occupation provides a mechanism to survive, to flourish, and to fulfill biological and social-cultural aspects of human needs (1). People with severe mental illness perceived employment to be meaningful, which also facilitated coping, self-management and maintenance of social contact, alleviated the impact of symptoms, and promoted recovery (2, 3). Additionally, those engaged in competitive work reported higher satisfaction with their finances and leisure activities, and showed improved self-esteem and symptoms (4). Employment provides not only the means to independent living and social integration, it also cultivates positive self-image, self-esteem and self-efficacy (5, 6), all of which promote recovery in people with mental illnesses. Being employed was also associated with a better quality of life in people with schizophrenia (7, 8).

Illness onset during adolescence and early adulthood (9) interferes with an individual’s vocational development such as education and employment (10). Among people with schizophrenia, the reported employment rate ranged from 10.2% in Norway to 30.3% in Germany (11–17). Employment rate was higher in developing countries, Asia (18, 19), and in rural areas (13, 18, 20). The employment rate was 67% in an urban area in India (19) and 77.6% in China, of which 93.9% were employed in rural areas and 26.7% in urban areas (18). In recent years, employment rates in schizophrenia were reported to be lower, which ranged from 19%-60% before 1990 to 4%-27% after 1990 in the UK (21), and decreased progressively from 57.9% in 1980 to 46.8% in 1995 in Singapore (22). The majority of people with psychosis were engaged in elementary jobs such as laborers, tradespersons, elementary clerical, sales, or service persons (13, 15, 20, 23).

The chronic relapsing nature of the illness can be disruptive to work. Being married, later onset of illness, absence of physical comorbidity, better neurocognitive functioning, lower severity of negative symptoms and depressive symptoms were reported to be associated with a higher likelihood of employment, while results on age, sex, ethnicity, education and positive symptoms were mixed (21, 24, 25). The majority of studies used a general psychiatric symptoms scale to assess symptoms of schizophrenia, which may not have captured all aspects of the construct of negative symptoms. The recent development and validation of negative symptoms measures refined the conceptualization of negative symptoms and allowed more in-depth investigation to be done. Our study had two objectives; first, we aimed to investigate the rate of employment and delineate vocational profiles in people with schizophrenia. Second, we aimed to examine demographic and clinical variables that were associated with employment. To better understand the associations between negative symptoms and employment, the Brief Negative Symptom Scale [BNSS (26)] was adopted in place of the negative symptoms factor in the Positive and Negative Syndrome Scale [PANSS (27)].

## Materials and Methods

### Study Setting and Participants

Community dwelling outpatients with schizophrenia were recruited from the Institute of Mental Health, Singapore, which provides comprehensive psychiatric care and rehabilitation services. Eligibility criteria included: age 21–65 years, a diagnosis of schizophrenia, ability to speak English and provide informed consent to participate in a research study. People with current alcohol or substance use disorder, history of brain injuries, neurological disorder or intellectual disability were excluded. Diagnosis of schizophrenia was ascertained using the Structured Clinical Interview for DSM-IV-TR Axis I Disorder-Patient Edition [SCID-I/P (28)]. Data were collected over 3.5 years (August 2014-December 2017). Ethics approval for the study was provided by the National Health Group’s Domain Specific Review Board.

### Assessments

Demographic and clinical information were collected. The Positive and Negative Syndrome Scale [PANSS (27)] was used to measure symptoms of schizophrenia with 30 items. Each item was rated from “1: Absent” to “7: Extreme”. PANSS factor analyses suggested a five-factor model (29, 30), which included positive symptoms, negative symptoms, excitement, depression and cognition/disorganization. The factor model suggested by a local validation study was adopted (29). Specifically, PANSS Positive was computed by summing ratings of PANSS items P1, P3, P6, and G9; PANSS Negative by summing items N2, N3, N4, N6, and G7; PANSS Excitement by summing items P4, P7, and G14; PANSS Depression by summing items G2, G3, and G6; and PANSS Cognition by summing items G10 and G12.

The Brief Negative Symptom Scale [BNSS (26)] measures severity of negative symptoms of schizophrenia with 13 items. Each item was rated from “0: No impairment” to “6: Severe deficit”. A second-order five-factor model was supported by factor analyses, in which Anhedonia, Asociality and Avolition belong to Motivation and Pleasure (MAP) factor and Blunted Affect and Alogia cluster into Emotional Expressivity (EE) factor (31, 32). Summation method was used to compute the factor scores. Factor scores from both BNSS MAP-EE model (MAP and EE) and BNSS five-factor model (Anhedonia, Asociality, Avolition, Blunted Affect, Alogia) were used in analyses.

The assessments were conducted by 3 trained raters, each with at least two years of experience in assessing people with schizophrenia. Intra-class correlation coefficient for BNSS and PANSS (>0.80) was established prior to study. Cases were discussed twice a month to ensure adequate agreement in ratings.

### The Coding of Employment Variables

Employment status, occupation, number of hours worked in the past week, monthly salary, and duration of work in the latest type of occupation were reported by participants. Hours worked was dichotomized according to the definition of part-time employment stipulated by the Singapore’s Ministry of Manpower (33, 34), i.e., less than 35 hours of work a week. Occupation of participants were coded according to the Singapore standard occupational classification (35) by two authors independently. The codes were then reviewed, disagreement was resolved by discussion and a final agreed code was assigned to the cases coded differently by the two raters. Cohen’s Kappa was computed to assess the strength of agreement.

### Statistical Analyses

Variables pertaining to employment, demographics, and clinical characteristics of the participants were summarized using descriptive statistics. χ^2^ test was used to test group differences between categorical variables or between categorical and ordinal variables. Mann-Whitney U test was used to test for group differences in the distributions of continuous variables that were not normally distributed. In the subgroup of employed participants, Spearman’s rank correlation with pairwise deletion was used to test the strength and direction of associations between employment outcomes and clinical variables; partial rank correlation with pairwise deletion was conducted to explore the association between employment outcomes and clinical variables after controlling for PANSS Positive, PANSS Cognition, or BNSS Avolition.

Logistic regression analyses were conducted with employment status as dependent variable, and age, sex, ethnicity, marital status, years of education, age of onset of psychotic symptoms, presence of current physical comorbidity, PANSS Positive, PANSS Excitement, PANSS Depression, PANSS Cognition, and either BNSS MAP and EE or BNSS Anhedonia, Asociality, Avolition, Blunted Affect and Alogia as independent variables. Another two logistic regression analyses on the subgroup of employed participants were conducted to examine variables associated with full-time/part-time employment, using the same independent variables.

## Results

### Descriptive Statistics and Vocational Profiles

Employment data from 276 participants was available. Two hundred seventy-four completed all clinical assessments. The demographic and clinical characteristics of the sample are presented in **Table 1**. One-hundred and twenty-seven (46.01%) participants were employed. The average PANSS and BNSS scores fell within the range of mild to moderate severity. The majority were single (n=214, 77.54%) and had at least secondary education (n=232, 84.06%). The most frequent physical comorbidity was those of endocrine, nutritional and metabolic diseases (n=130, 47.10%), followed by diseases of circulatory system (n=54, 19.57%), diseases of skin (n=24, 8.70%), diseases of musculoskeletal system or connective tissue (n=21, 7.61%), diseases of respiratory system (n=17, 6.16%), and diseases of genitourinary system (n=16, 5.80%).

**Table 1:** Demographic and clinical characteristics of participants.

Variable	Mean (SD) or n (%)		
	All	Employed	Unemployed
Sex (Male)	152 (55.07%)	68 (53.54%)	84 (56.38%)
Age (year)	40.42 (10.14)	39.84 (9.84)	40.91 (10.39)
Age of onset of psychotic symptoms	23.11 (6.50)	23.41 (6.55)	22.85 (6.46)
Antipsychotic dose^†^	461.67 (406.25)	423.08 (378.82)	494.57 (426.77)
Current physical comorbidity (yes)	178 (64.49%)	72 (56.69%)	106 (71.14%)
Ethnicity			
Chinese	233 (84.42%)	104 (81.89%)	129 (86.58%)
Malay	20 (7.25%)	10 (7.87%)	10 (6.71%)
Indian	22 (7.97%)	12 (9.45%)	10 (6.71%)
Others	1 (0.36%)	1 (0.79%)	–
Marital Status			
Never Married	214 (77.54%)	95 (74.80%)	119 (79.87%)
Married	36 (13.04%)	20 (15.75%)	16 (10.74%)
Separated/Divorced	24 (8.70%)	11 (8.66%)	13 (8.72%)
Widowed	2 (0.72%)	1 (0.79%)	1 (0.67%)
Total Years of Education	13.17 (3.12)	13.48 (2.96)	12.90 (3.23)
Education level			
Lower than Primary	5 (1.81%)	2 (1.57%)	3 (2.01%)
Primary	39 (14.13%)	16 (12.60%)	23 (15.44%)
Secondary	60 (21.74%)	27 (21.26%)	33 (22.15%)
Pre-University	20 (7.25%)	8 (6.30%)	12 (8.05%)
Certificate/Vocational	52 (18.84%)	24 (18.90%)	28 (18.79%)
Diploma	71 (25.72%)	32 (25.20%)	39 (26.17%)
Degree and above	29 (10.51%)	18 (14.17%)	11 (7.38%)
PANSS Total score	58.11 (12.88)	56.42 (12.85)	59.55 (12.77)
PANSS Positive	8.31 (4.35)	7.82 (4.23)	8.72 (4.43)
PANSS Negative	11.28 (4.05)	10.82 (4.01)	11.68 (4.05)
PANSS Excitement	4.60 (2.06)	4.63 (1.88)	4.57 (2.20)
PANSS Depression	5.60 (2.50)	5.53 (2.41)	5.66 (2.58)
PANSS Cognition	4.34 (1.64)	4.33 (1.73)	4.34 (1.57)
BNSS Total score	24.92 (11.88)	21.4 (10.88)	27.91 (11.91)
Anhedonia	7.09 (3.48)	6.08 (3.47)	7.96 (3.27)
Asociality	2.82 (1.79)	2.63 (1.82)	2.99 (1.75)
Avolition	4.58 (2.17)	3.54 (1.97)	5.46 (1.92)
Blunted Affect	5.43 (4.54)	4.63 (3.94)	6.10 (4.91)
Alogia	4.10 (3.53)	3.74 (3.39)	4.41 (3.64)
Motivation and Pleasure (MAP)	14.49 (6.35)	12.25 (6.29)	16.41 (5.76)
Emotional Expressivity (EE)	9.53 (7.13)	8.37 (6.37)	10.51 (7.60)

^†^Antipsychotic doses were converted into total daily chlorpromazine equivalents.

PANSS, Positive and Negative Syndrome Scale; BNSS, Brief Negative Symptom Scale.

Details of employment are presented in **Table 2**. The strength of agreement on occupation categorization between the two independent raters was almost perfect, kappa=0.878 (CI=0.813-0.943), *p*<0.001 (36). Among those who were unemployed, 4 (2.68%) were students and 7 (4.70%) reported themselves as homemakers. Three (2.01%) out of 4 (2.68%) who reported to have retired had not reached the retirement age (62 years old) stipulated by the Retirement and Re-employment Act in Singapore (34).

**Table 2:** Description of employment.

Variable	Mean (SD) or n (%)
Unemployed	149 (53.99%)
Homemaker	7 (4.70%)
Student	4 (2.68%)
Retired	4 (2.68%)
Job preparatory program/Job Club	18 (12.08%)
In between jobs	7 (4.70%)
Unemployed	109 (73.15%)
Employed	127 (46.01%)
Supported employment/Job Club	14 (11.02%)
Employed by family	5 (3.94%)
Competitive employment	108 (85.04%)
Type of occupation	
Senior manager	1 (0.79%)
Professionals	4 (3.15%)
Associate professionals	30 (23.62%)
Clerical support workers	15 (11.81%)
Service and sales workers	38 (29.92%)
Craftsmen and related workers	2 (1.57%)
Plant and machine operators and assemblers	3 (2.36%)
Cleaners and laborers	34 (26.77%)
Duration of work in the latest type of occupation (year)	5.16 (6.09)
Part-time employment	62 (48.82%)
No. of hours worked per week	33.64 (17.49)
Part-timers	18.82 (9.52)
Full-timers	47.77 (10.03)
Monthly salary (SGD)	1221.28 (1038.89)
20 percentile	450.00
50 percentile	1000.00
Monthly salary: Full-time (SGD)	1805.28 (1074.46)
20 percentile	1000.00
50 percentile	1550.00
Monthly salary: Part-time (SGD)	577.89 (460.04)
20 percentile	215.00
50 percentile	490.00

Employment status was not significantly different by sex, *χ*^2^(1)=0.222, *p*=0.637; ethnicity, *χ*^2^(3)=2.124, *p*=0.547, and marital status, *χ*^2^(3)=1.559, *p*=0.669. The differences between the employed and unemployed on age (*U*=8933.50, *p*=0.424), age of onset of psychotic symptoms (*U*=8927.50, *p*=0.418), antipsychotic dose (*U*=8308.00, *p*=0.081) and total years of education (*U*=8255.50, *p*=0.068) were not significant. Presence of current physical comorbidity was significantly associated with employment status, *χ*^2^(1)=6.250, *p*=0.012.

Eighteen (62.07%) individuals who had at least a degree were employed, while 109 (44.13%) individuals with lower educational attainment were employed, *χ*^2^(1)=3.362, *p*=0.067. Occupation was significantly different by educational attainment, *χ*^2^(18)=42.913, *p*=0.001. The majority worked in occupation compatible with their education, but some worked in occupations requiring less than their educational attainment. For example, no individual with primary and below education worked as managers/professionals/associate professionals, while 12 (66.67%) of them were craftsmen/machine operators/cleaners/laborers; 24 (48.00%) individuals with at least a diploma were managers/professionals/associate professionals; while 8 (16.00%) were craftsmen/machine operators/cleaners/laborers.

Participation in psychosocial rehabilitation was related to unemployment, *χ*^2^(1)=4.988, *p*=0.026. Among the unemployed, 47 (31.76%) were engaged in psychosocial rehabilitation programs; of the 47, 18 (38.30%) were engaged in job preparatory programs (37, 38). Among the employed, 25 (19.84%) were engaged in psychosocial rehabilitation programs, of which 14 (56.00%) were under supported employment.

There were significant differences in proportions of full-time employment in different occupations, *χ*^2^(3)=14.620, *p*=0.002. Twenty-three (65.71%) of the managers/professionals/associate professionals worked full-time, 25 (65.79%) service and sales workers worked full-time, while only 5 (33.33%) clerical support workers and 12 (30.77%) craftsmen/machine operators/cleaners/laborers worked full-time.

### Factors Associated With Employment Status

In the model with BNSS MAP-EE, higher symptom severity on the BNSS MAP (*OR*=0.897, CI=0.854-0.941) and having current physical comorbidity (*OR*=0.533, CI=0.304-0.937) were associated with a lower likelihood of employment. In the model with BNSS five-factor, Avolition (*OR*=0.541, CI=0.440-0.666) and PANSS Positive (*OR*=0.924, CI=0.855-0.997) were associated with lower likelihood of employment (see **Table 3**).

**Table 3:** Logistic Regression of factors associated with employment status.

	Model with MAP-EE				Model with BNSS 5 factors			
	B	S.E.	Exp(B)	Sig.	B	S.E.	Exp(B)	Sig.
Age	-0.006	0.015	0.994 (0.965 - 1.024)	0.693	-0.010	0.016	0.990 (0.959 - 1.023)	0.550
Age of onset of psychotic symptoms	0.022	0.022	1.022 (0.978 - 1.068)	0.335	0.004	0.024	1.004 (0.959 - 1.052)	0.851
Sex (Female)	-0.145	0.280	0.865 (0.500 - 1.499)	0.606	-0.003	0.301	0.997 (0.553 - 1.799)	0.992
Ethnicity (Malay)^†^	0.190	0.503	1.209 (0.451 - 3.241)	0.705	0.317	0.553	1.373 (0.464 - 4.061)	0.566
Ethnicity (Indian)^†^	0.592	0.509	1.807 (0.666 - 4.903)	0.245	0.721	0.561	2.057 (0.685 - 6.178)	0.199
Marital Status (Married)	0.239	0.418	1.270 (0.559 - 2.883)	0.568	0.180	0.458	1.198 (0.488 - 2.940)	0.694
Education (year)	0.038	0.043	1.038 (0.954 - 1.131)	0.385	0.045	0.046	1.046 (0.957 - 1.144)	0.321
Current physical comorbidity	-0.629	0.288	0.533 (0.304 - 0.937)	0.029	-0.514	0.312	0.598 (0.325 - 1.102)	0.099
PANSS Positive	-0.046	0.035	0.955 (0.891 - 1.023)	0.192	-0.079	0.039	0.924 (0.855 - 0.997)	0.043
PANSS Excitement	0.032	0.070	1.032 (0.899 - 1.185)	0.653	0.061	0.075	1.063 (0.917 - 1.233)	0.417
PANSS Depression	0.031	0.060	1.032 (0.918 - 1.160)	0.598	0.047	0.066	1.048 (0.920 - 1.193)	0.484
PANSS Cognition	0.152	0.094	1.164 (0.968 - 1.399)	0.107	0.161	0.105	1.174 (0.956 - 1.442)	0.125
BNSS MAP	-0.109	0.025	0.897 (0.854 - 0.941)	<0.001				
BNSS EE	-0.013	0.022	0.987 (0.946 - 1.030)	0.537				
BNSS Anhedonia					0.030	0.061	1.030 (0.913 - 1.162)	0.630
BNSS Asociality					0.163	0.099	1.177 (0.969 - 1.429)	0.101
BNSS Avolition					-0.615	0.106	0.541 (0.440 - 0.666)	<0.001
BNSS Blunted Affect					-0.049	0.040	0.952 (0.881 - 1.030)	0.220
BNSS Alogia					0.044	0.055	1.045 (0.939 - 1.164)	0.422

^†^Reference group is Chinese ethnicity.

PANSS, Positive and Negative Syndrome Scale; BNSS, Brief Negative Symptom Scale; MAP, Motivation and Pleasure; EE, Emotional Expressivity.

### Factors Associated With Full-Time Employment

More severe Avolition (*OR*=0.644, CI=0.475-0.872) and female gender (*OR*=0.286, CI=0.128-0.637 in the model with BNSS MAP-EE; *OR*=0.289, CI=0.126-0.662 in the model with BNSS five-factor) were significantly associated with lower likelihood of working full-time. All other BNSS factors and variables were not significantly associated with full-time employment (**Table 4**).

**Table 4:** Logistic Regression of factors associated with full-time employment.

	Model with MAP-EE				Model with BNSS 5 factors			
	B	S.E.	Exp(B)	Sig.	B	S.E.	Exp(B)	Sig.
Age	-0.042	0.026	0.959 (0.912 - 1.009)	0.108	-0.046	0.027	0.955 (0.905 - 1.008)	0.092
Age of onset of psychotic symptoms	0.063	0.036	1.065 (0.992 - 1.144)	0.083	0.061	0.039	1.063 (0.984 - 1.148)	0.122
Sex (Female)	-1.253	0.409	0.286 (0.128 - 0.637)	0.002	-1.243	0.424	0.289 (0.126 - 0.662)	0.003
Ethnicity (Malay)^†^	0.655	0.733	1.925 (0.458 - 8.096)	0.372	1.020	0.822	2.773 (0.554 - 13.878)	0.214
Ethnicity (Indian)^†^	-0.051	0.679	0.950 (0.251 - 3.592)	0.940	0.059	0.772	1.061 (0.233 - 4.821)	0.939
Marital Status (Married)	0.687	0.586	1.987 (0.630 - 6.269)	0.241	0.649	0.646	1.914 (0.540 - 6.790)	0.315
Education (year)	-0.052	0.070	0.950 (0.828 - 1.088)	0.457	-0.043	0.074	0.958 (0.828 - 1.108)	0.561
Current physical comorbidity	0.186	0.434	1.204 (0.515 - 2.817)	0.668	0.244	0.454	1.276 (0.524 - 3.107)	0.591
PANSS Positive	0.018	0.056	1.018 (0.912 - 1.137)	0.750	-0.011	0.062	0.989 (0.876 - 1.116)	0.853
PANSS Excitement	-0.169	0.123	0.845 (0.664 - 1.076)	0.172	-0.147	0.133	0.863 (0.665 - 1.120)	0.268
PANSS Depression	0.081	0.093	1.085 (0.904 - 1.302)	0.382	0.107	0.101	1.112 (0.913 - 1.355)	0.289
PANSS Cognition	0.057	0.138	1.059 (0.808 - 1.388)	0.678	0.077	0.147	1.080 (0.810 - 1.440)	0.600
BNSS MAP	-0.014	0.035	0.986 (0.921 - 1.057)	0.695				
BNSS EE	-0.023	0.037	0.977 (0.909 - 1.051)	0.537				
BNSS Anhedonia					0.111	0.095	1.118 (0.927 - 1.347)	0.242
BNSS Asociality					0.165	0.156	1.180 (0.870 - 1.600)	0.288
BNSS Avolition					-0.440	0.155	0.644 (0.475 - 0.872)	0.004
BNSS Blunted Affect					-0.060	0.070	0.942 (0.821 - 1.080)	0.390
BNSS Alogia					0.032	0.084	1.033 (0.876 - 1.219)	0.701

^†^Reference group is Chinese ethnicity.

PANSS, Positive and Negative Syndrome Scale; BNSS, Brief Negative Symptom Scale; MAP, Motivation and Pleasure; EE, Emotional Expressivity.

### Association Between Employment Outcomes and Clinical Variables

Spearman’s correlations between employment outcomes and clinical variables and partial rank correlations between the same variables after controlling for PANSS Positive or PANSS Cognition are presented in **Table 5**. Among PANSS factors, PANSS Negative and PANSS Cognition had the strongest correlation with monthly salary (PANSS Negative: *ρ*=-0.277, *p*=0.002; PANSS Cognition: *ρ*=-0.246, *p*=0.006) and hourly salary (PANSS Negative: *ρ*=-0.228, *p*=0.011; PANSS Cognition: *ρ*=-0.316, *p*<0.001). Among BNSS factors, Avolition had the strongest correlation with monthly salary (*ρ*=-0.334, *p*<0.001) and hours worked (*ρ*=-0.270, *p*=0.002) after partialling out the effect of PANSS Positive, and with monthly salary (*ρ*=-0.292, *p*=0.001) and hours worked (*ρ*=-0.280, *p*=0.002) after controlling for PANSS Cognition.

**Table 5:** Non-parametric correlation between work outcomes and clinical variables.

Variables	Spearman’s rank correlation (zero order correlation)				Partial correlation controlling for PANSS Positive				Partial correlation controlling for PANSS Cognition			
	Monthly Salary	Hourly Salary	Hours worked	Work duration	Monthly Salary	Hourly Salary	Hours worked	Work duration	Monthly Salary	Hourly Salary	Hours worked	Work duration
Hourly Salary	0.737^**^	1.000	0.208^*^	0.382^**^	0.733^**^	1.000	0.209^*^	0.363^**^	0.717^**^	1.000	0.217^*^	0.348^**^
Hours worked (past week)	0.766^**^	0.208^*^	1.000	0.213^*^	0.770^**^	0.209^*^	1.000	0.215^*^	0.789^**^	0.217^*^	1.000	0.216^*^
Work duration in the line	0.392^**^	0.382^**^	0.213^*^	1.000	0.380^**^	0.363^**^	0.215^*^	1.000	0.365^**^	0.348^**^	0.216^*^	1.000
**PANSS factors**												
PANSS Total	-0.342^**^	-0.376^**^	-0.109	-0.230^**^	-0.360^**^	-0.365^**^	-0.142	-0.150	-0.248^**^	-0.239^**^	-0.136	-0.152
PANSS Positive	-0.120	-0.167	-0.009	-0.178^*^	–	–	–	–	-0.043	-0.070	-0.007	-0.128
PANSS Negative	-0.277^**^	-0.228^*^	-0.172	-0.111	-0.261^**^	-0.203^*^	-0.173	-0.080	-0.187^*^	-0.091	-0.193^*^	-0.028
PANSS Excitement	-0.092	-0.072	-0.092	-0.004	-0.058	-0.023	-0.094	0.054	-0.053	-0.020	-0.092	0.028
PANSS Depression	-0.063	-0.113	-0.043	-0.135	-0.021	-0.058	-0.043	-0.077	-0.090	-0.153	-0.044	-0.156
PANSS Cognition	-0.246^**^	-0.316^**^	-0.006	-0.180^*^	-0.220^*^	-0.280^**^	-0.003	-0.131	–	–	–	–
**BNSS factors**												
BNSS Total	-0.238^**^	-0.283^**^	-0.029	-0.111	-0.223^*^	-0.264^**^	-0.028	-0.086	-0.150	-0.171	-0.030	-0.037
BNSS MAP	-0.209^*^	-0.152	-0.084	-0.012	-0.195^*^	-0.130	-0.084	0.015	-0.150	-0.069	-0.086	0.041
BNSS EE	-0.180^*^	-0.289^**^	0.003	-0.170	-0.165	-0.271^**^	0.004	-0.148	-0.087	-0.181^*^	0.006	-0.106
BNSS Anhedonia	-0.129	-0.118	-0.003	0.014	-0.118	-0.102	-0.002	0.033	-0.081	-0.055	-0.001	0.054
BNSS Asociality	-0.090	-0.050	0.011	0.001	-0.060	-0.006	0.013	0.051	-0.018	0.048	0.013	0.058
BNSS Avolition	-0.340^**^	-0.199^*^	-0.270^**^	-0.055	-0.334^**^	-0.188^*^	-0.270^**^	-0.041	-0.292^**^	-0.122	-0.280^**^	-0.006
BNSS Blunted Affect	-0.185^*^	-0.267^**^	-0.012	-0.190^*^	-0.166	-0.242^**^	-0.011	-0.161	-0.115	-0.183^*^	-0.011	-0.141
BNSS Alogia	-0.111	-0.214^*^	0.024	-0.085	-0.105	-0.207^*^	0.025	-0.076	-0.010	-0.095	0.029	-0.011
**BNSS items**												
1. Intensity of pleasure	-0.075	-0.100	0.031	0.019	-0.067	-0.089	0.032	0.033	-0.049	-0.068	0.032	0.040
2. Frequency of pleasurable activities	-0.201^*^	-0.129	-0.078	-0.017	-0.188^*^	-0.109	-0.077	0.007	-0.160	-0.072	-0.078	0.019
3. Intensity of expected pleasure	-0.060	-0.088	0.056	0.052	-0.051	-0.076	0.057	0.067	0.006	-0.005	0.059	0.105
4. Distress	0.002	-0.096	0.129	-0.076	-0.029	-0.143	0.131	-0.125	0.040	-0.052	0.131	-0.051
5. Asociality: Behaviour	-0.095	-0.075	0.024	0.014	-0.062	-0.028	0.028	0.071	-0.011	0.037	0.027	0.082
6. Asociality: Internal experience	-0.067	-0.024	0.002	-0.016	-0.045	0.009	0.003	0.019	-0.017	0.045	0.003	0.022
7. Avolition: Behaviour	-0.373^**^	-0.201^*^	-0.356^**^	-0.023	-0.372^**^	-0.198^*^	-0.356^**^	-0.017	-0.330^**^	-0.130	-0.367^**^	0.025
8. Avolition: Internal experience	-0.253^**^	-0.163	-0.156	-0.067	-0.244^**^	-0.149	-0.156	-0.051	-0.205^*^	-0.092	-0.160	-0.024
9. Facial Expression	-0.204^*^	-0.288^**^	-0.014	-0.153	-0.192^*^	-0.273^**^	-0.013	-0.134	-0.159	-0.236^**^	-0.013	-0.118
10. Vocal Expression	-0.147	-0.163	-0.054	-0.131	-0.135	-0.148	-0.053	-0.114	-0.083	-0.081	-0.054	-0.085
11. Expressive Gestures	-0.116	-0.229^*^	0.045	-0.210^*^	-0.088	-0.195^*^	0.049	-0.172	-0.039	-0.141	0.049	-0.163
12. Quantity of Speech	-0.132	-0.219^*^	0.005	-0.082	-0.129	-0.217^*^	0.006	-0.078	-0.031	-0.098	0.009	-0.006
13. Spontaneous Elaboration	-0.098	-0.206^*^	0.028	-0.089	-0.092	-0.199^*^	0.029	-0.080	-0.001	-0.094	0.033	-0.020

*. Correlation is significant at the 0.05 level (2-tailed).

**. Correlation is significant at the 0.01 level (2-tailed).

PANSS, Positive and Negative Syndrome Scale; BNSS, Brief Negative Symptom Scale; MAP, Motivation and Pleasure; EE, Emotional Expressivity.

When the effect of BNSS Avolition was adjusted for, the correlation between hourly salary and hours worked became smaller and non-significant (*ρ*=0.163, *p*=0.071). Most other correlations decreased very slightly (hourly salary and work duration: *ρ*=0.379, *p*<0.001; monthly salary and hourly salary: *ρ*=0.726, *p*<0.001; monthly salary and hours worked: *ρ*=0.744, *p*<0.001; hours worked and work duration: *ρ*=0.206, *p*=0.021), while correlation between monthly salary and work duration increased very slightly (*ρ*=0.398, *p*<0.001).

## Discussion

In the present investigation, 46.01% of the participants with schizophrenia were employed with almost half of them working part-time. Negative symptoms, specifically MAP/Avolition, positive symptoms, and current physical comorbidity were associated with likelihood of employment.

The rate of employment in our sample is higher than the rates reported in previous studies in developed countries (11–17), but lower than the rate reported in an urban area in India (19). In general, Singapore has a low unemployment rate (39, 40) and Warner (41) had proposed that employment rate in people with schizophrenia may be associated with the prevailing economic condition and unemployment rate in the general population. Nevertheless, the employment rate in our study was lower than the rate reported in the Singapore population, which ranged between 79.70%–80.70% in people aged 25–64 in years 2014–2017 (33, 34).

Similar to past studies (13, 20, 23), the majority were elementary or support workers, only about a quarter were managers/professionals/associate professionals. This proportion was much lower compared to the Singapore population in 2017, where 11.74% were engaged in managerial positions, 20.03% in professional jobs, and 20.73% were associate professionals or technicians. More participants were engaged in service and sales related jobs (sample: 29.92%; population in 2017: 11.59%), and cleaner and laborer related jobs (sample: 26.77%; population in 2017: 7.38%). It was postulated that people with schizophrenia tend to engage in work that requires less social interaction (42). Cognitive impairment associated with schizophrenia (43) and lower educational attainment (10) may also limit the career options for people with mental illnesses. The availability of jobs suitable for this cohort which are less cognitively demanding and less communication intensive may be limited in an urban city, which may also explain the lower employment rate in urban areas (18) and in the recent decades (22) due to urbanization.

Though the average hours worked a week by people with schizophrenia working full-time were seemingly equivalent to the population in 2017 (sample: 47.77 hours; population: 45.9 hours), the median monthly salary (sample: $1,550; population: $3,749) and 20^th^ percentile monthly salary (sample: $1,000; population: $2,000) from full-time work was lower in the sample (33, 34). This might be because the majority of participants were engaged in elementary jobs which offered lower pay. There was evidence that people with psychiatric disorders earned much lesser than those without mental disorders (44–46). A study in Finland also showed that more than half of the people with schizophrenia at working age did not have earning or earned very little (47). Full-timer in sales/services sectors who worked longer hour (51.14 hours, SD=12.49) constituted a large proportion of the employed sample, which may explain the longer average hours worked in our sample.

Our finding on association of negative symptoms and employment status in schizophrenia is not novel (8, 19, 24). Negative symptoms were also suggested to be associated with vocational impairment (48) and reduced work performance (49). However, the implications were limited by the non-discerning singular measure of negative symptoms. Knowing the types of negative symptoms crucial to employment status provides opportunity to enhance treatment regime, but existing evidence investigating the association between separate domains of negative symptoms conceptualization and employment status was scarce. Using the Scale for the Assessment of Negative Symptoms (SANS), Llerena et al. (50) demonstrated the association between experiential symptoms, i.e., apathy/avolition and anhedonia/asociality, and employment in schizophrenia and schizoaffective patients who participated in supported employment programs, but the association became non-significant after age was adjusted. They also showed that Avolition, relative to Anhedonia/Asociality, was associated with employment status, but the impact of other important covariates was not controlled in this analysis. Using a contemporary negative symptom scale, our study demonstrated that MAP and Avolition were associated with employment status after controlling for the impact of important covariates related to employment in community dwelling outpatients with schizophrenia. Avolition was also uniquely associated with working part-time after controlling for employment-related covariates and correlated with hours worked after adjusting for PANSS Positive and PANSS Cognition respectively, suggesting the importance of Avolition in both working and time spent working. Our study also suggested that although MAP was associated with employment status, it’s not associated with working part-time, hours worked and work duration, as also implied by the small or negligible associations of Anhedonia and Asociality with work outcome variables. The fact that the association between hourly salary and hours worked become weaker and non-significant after controlling for BNSS Avolition also suggested Avolition may be a common factor explaining the associations. Furthermore, only the behavioral aspect of Avolition was associated with salary and hours worked, suggesting inner motivation alone may not be sufficient, more research about transitioning inner motivation to behavior and policies alleviating barriers to work might help to encourage higher engagement in work.

Though a diagnosis of schizophrenia and presence of psychosis were associated with work functioning (51), few studies found association between positive symptoms and functioning (52). Results on association of positive symptoms and employment status were mixed (21). In our study, higher positive symptoms were associated with lower likelihood of employment only in the model with BNSS five factors, but the effect size was small. In literatures with schizophrenia-spectrum disorders, the employed patients had better positive symptoms scores (53), while unemployed patients who remained unemployed at 3-year follow-up had more severe positive symptoms than those who obtained a job (54). Positive symptoms were also significantly correlated with all domains of disability in people with schizophrenia (55). Maybe it is the persistent and severe positive symptoms that deter people from employment (51), therefore the impact of positive symptoms may not be apparent in our sample comprising mostly people with mild to moderate positive symptoms. On the other hand, a study showed that the employed schizophrenia outpatients had slightly worse PANSS Positive at 18-month follow-up, while the change in PANSS Positive in the unemployed outpatients was not statistically significant (56). While the change may not be clinically significant, the impact of employment on symptoms should be scrutinized in a carefully designed study in the future, as this could be one of the reasons of the small effect size in our study and inconsistent results on association of positive symptoms and employment status observed in the literature. The relationship between positive symptoms and employment could be complicated, non-linear and vary at different time points depending on environmental stimuli including employment and work environment. PANSS Positive was negatively associated with duration of work in our study, suggesting that severity of positive symptoms may be an important determinant in work perseverance. Although the correlation between PANSS Positive and work duration decreased and became non-significant after controlling for PANSS Cognition, the fact that the correlation between PANSS Positive and PANSS Cognition was non-negligible (*ρ*=0.253, *p*<0.001) and PANSS Cognition was also associated with duration of work, it is possible that both positive symptoms and cognitive symptoms may account for work perseverance.

More severe PANSS Cognition was associated with lower salary and shorter work duration. Although PANSS Cognition is only a proxy of cognition construct, the results were in line with previous studies that cognition was not associated with employment status but associated with work tenure (57, 58). Cognitive programs added to work rehabilitation program were also associated with higher earnings (59, 60). Additionally, more severe BNSS EE, BNSS Blunted Affect and BNSS Alogia were also found to be associated with lower salary. BNSS EE and its domains were shown to be associated with cognition (32, 61). A common cognitive factor might partially explain the associations between BNSS EE and salary, which was evidenced by their weaker associations after controlling for PANSS Cognition.

Individuals with schizophrenia were more likely to have physical comorbidity (62, 63). Our results were consistent with previous findings that the presence of physical comorbidity was associated with unemployment status (64) and lower likelihood of competitive employment in people with psychiatric disabilities (65). Having physical problems was also identified as one of the first five barriers to employment for both the employed and unemployed individuals with severe mental illness (66).

Female sex was associated with part-time employment, consistent with the report on lower labor force participation rate and income from work among females in the population (33, 34). Our results on sex difference in working part-time may be a reflection of employment patterns of female in the society in general, which might partially be due to the assumed family role of females (67–69).

Educational attainment was associated with occupation, but there were observations of people working in occupation not on par with their educational attainment. This is consistent with findings suggesting significant decline in vocational status in people with schizophrenia, and that people who started with higher status jobs had more room to decline (70). Additionally, educational attainment had at most marginal association with employment status. Contrary to previous finding that having a degree was associated with higher likelihood of employment in schizophrenia (13), the non-significant association in our study suggested that other factors may have larger impact on employment status. Attitudes and value about work, tolerance and stigma against mentally ill patients, social and family support, provision of rehabilitation services, economic burden, social security and benefit systems, and employment legislation (8, 13, 16, 19, 23, 66, 71, 72) would be associated with employment in people with severe mental illness.

A few limitations need to be considered in understanding the results. First, the study recruited from community dwelling outpatients with the opportunity to seek employment, the results cannot be generalized to all schizophrenia patients. The severely ill and institutionalized, who are unable to provide informed consent, remain under-surveyed. The cross-sectional nature of the study did not allow causal relationships to be drawn. Social desirability bias may affect how the participants describe their employments and might lead to inflation of their employment data. Further, the reported total number of hours worked in the past week may be susceptible to recall bias, and may not fully represent hours worked in a typical week. Additionally, some patients who were presently unemployed might be transitioning to employment. Nonetheless, the reported information still provided a proxy of the examined constructs; people with psychiatric illness were also shown to be able to report these information without much deviation (73, 74).

To conclude, our study reported the employment rate and the characteristics of employment in community dwelling individuals with schizophrenia in an Asian metropolitan. Our results indicated that negative symptoms and presence of physical comorbidity were associated with lower likelihood of employment, while positive symptoms may have small impact on lower likelihood of employment. Our findings suggested that identification and effective management of these factors might improve employment opportunities and facilitate meaningful vocational engagement for individuals with schizophrenia. Vocational rehabilitation programs should take these factors into consideration to maximize the chance of employment.

## Data Availability Statement

The participants of this study did not agree for their data to be shared publicly, so supporting data is not publicly available. Further enquiries could be directed to either the corresponding author or to imhresearch@imh.com.sg.

## Ethics Statement

The studies involving human participants were reviewed and approved by National Health Group’s Domain Specific Review Board. The patients/participants provided their written informed consent to participate in this study.

## Author Contributions

GR and JL conceived the research project and wrote the research protocol. GR and MSA conducted data collection. MSA conducted literature review, data analyses, and wrote the first draft of the manuscript. GR and JL gave substantial comments and edited the manuscript. All authors contributed to the article and approved the submitted version.

## Funding

This study is supported by the Singapore Ministry of Health’s National Medical Research Council under the Centre Grant Programme (Grant No.: NMRC/CG/004/2013).

## Conflict of Interest

The authors declare that the research was conducted in the absence of any commercial or financial relationships that could be construed as a potential conflict of interest.
